# SOX5 predicts poor prognosis in lung adenocarcinoma and promotes tumor metastasis through epithelial-mesenchymal transition

**DOI:** 10.18632/oncotarget.22443

**Published:** 2017-11-06

**Authors:** Xin Chen, Yufei Fu, Hongfei Xu, Peng Teng, Qiong Xie, Yiran Zhang, Caochong Yan, Yiqiao Xu, Chunqi Li, Jianying Zhou, Yiming Ni, Weidong Li

**Affiliations:** ^1^ Department of Thoracic and Cardiovascular Surgery, The First Affiliated Hospital of Zhejiang University, Hangzhou, PR China; ^2^ Zhejiang Key Laboratory of Gastro-Intestinal Pathophysiology, Zhejiang Hospital of Traditional Chinese Medicine, First Affiliated Hospital of Zhejiang Chinese Medical University, Hangzhou, PR China; ^3^ Department of Respiratory Disease, The First Affiliated Hospital, Zhejiang University, Hangzhou, PR China; ^4^ Hunter Biotechnology, Inc., Hangzhou, PR China

**Keywords:** SOX5, EMT, lung adenocarcinoma, prognosis

## Abstract

Lung cancer is the leading cause of cancer-related death worldwide. Epithelial-mesenchymal transition (EMT) promotes lung cancer progression and metastasis, especially in lung adenocarcinoma. Sex determining region Y-box protein 5 (SOX5) is known to stimulate the progression of various cancers. Here, we used immunohistochemical analysis to reveal that SOX5 levels were increased in 90 lung adenocarcinoma patients. The high SOX5 expression in lung adenocarcinoma and non-tumor counterparts correlated with the patients’ poor prognosis. Inhibiting SOX5 expression attenuated metastasis and progression in lung cancer cells, while over-expressing SOX5 accelerated lung adenocarcinoma progression and metastasis via EMT. An *in vivo* zebrafish xenograft cancer model also showed *SOX5* knockdown was followed by reduced lung cancer cell proliferation and metastasis. Our results indicate SOX5 promotes lung adenocarcinoma tumorigenicity and can be a novel diagnosis and prognosis marker of the disease.

## INTRODUCTION

Lung cancer is the leading cause of cancer-related death worldwide, with the most common pathologic type being lung adenocarcinoma (LAC) [1, 2]. In spite of the recent progress in targeted therapy, most LAC patients eventually died due to recurrence and drug resistance [3, 4]. These poor outcomes are due to the shortage of a better molecular biomarker for prognosis estimation. Identification of reliable prognostic predictors which can improve diagnosis, prognostic stratification, and serve as possible therapeutic targets is needed in LAC.

Metastasis via epithelial to mesenchymal transition (EMT) is a distinguishing feature of tumor development in most cancers [5–7]. EMT is a developmental process in which epithelial cells lose their epithelial features and develop a mesenchymal phenotype. In LAC, abnormal activation of EMT leads to tumor invasion, metastatic dissemination, and acquisition of therapeutic resistance, coupled with poor prognosis [8, 9]. EMT is characterized by loss of epithelial makers (e.g. E-cadherin), up-regulation of mesenchymal markers (e.g. Vimentin) and smooth muscle actin (SMA), acquisition of fibroblast-like morphology with cytoskeleton reorganization, and increases in motility, invasiveness, and metastatic capabilities [6, 10–14].

Sex determining region Y-box protein 5 (SOX5) expression is correlated with various cancers including prostate tumor, breast cancer, glioma, hepatocellular carcinoma and nasopharyngeal carcinoma [15–19]. Tumors consist not only of malignant cancer cells, but also stromal cells that support the tumor microenvironment. These include fibroblasts and immune cells [20, 21], as well as endothelial cells and smooth muscle cells that form blood vessels and provide nourishment to the tumor [22]. In this study, the function analysis of the transcription factor SOX5 was not only studied in tumors but also in the tumor microenvironment. We analyzed 90 LAC cases by tissue microarray technology to see if SOX5 could serve as a biomarker for LAC prognosis and a therapeutic target to combat LAC metastasis.

## RESULTS

### SOX5 is over-expressed in LAC cells and tissues

**Figure 1:**
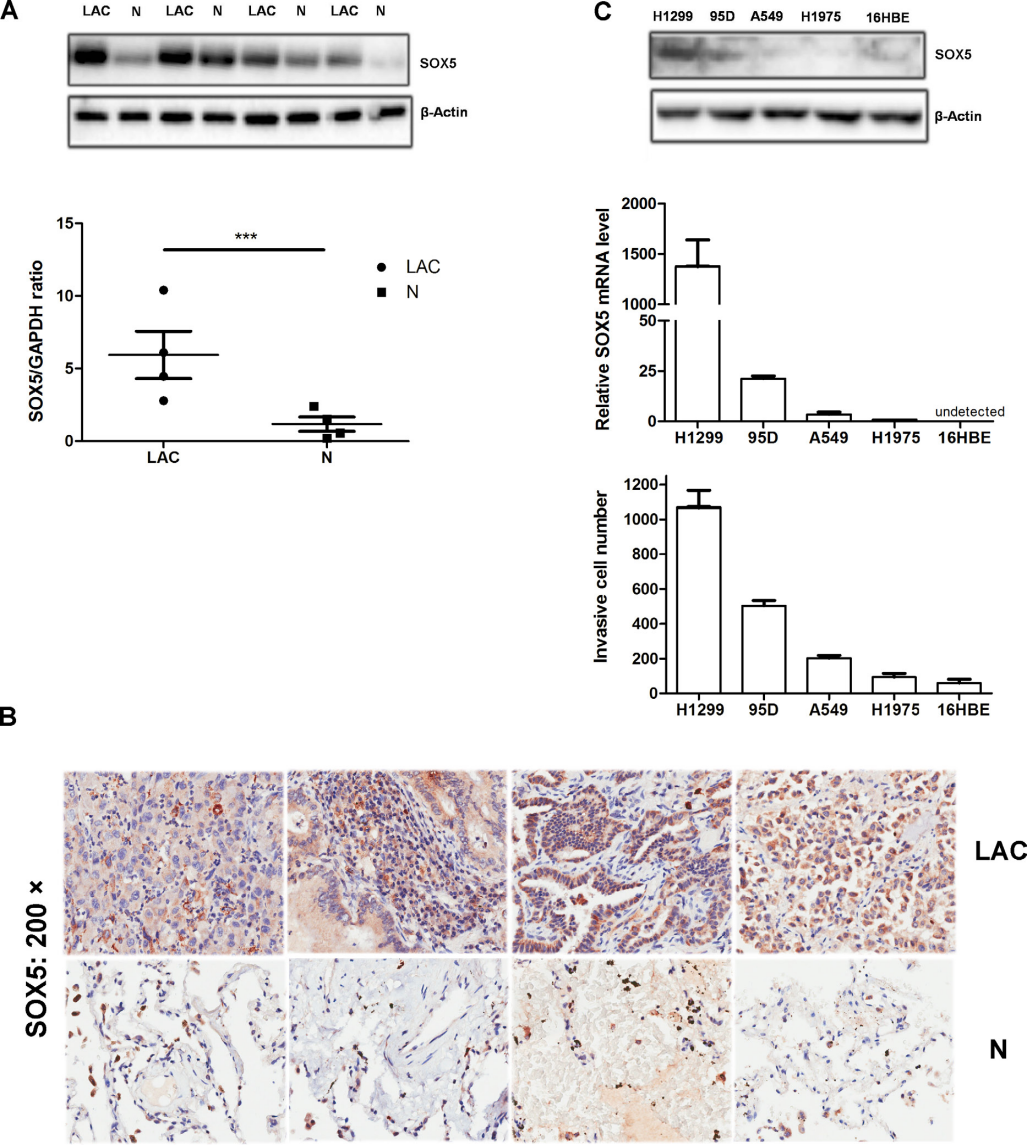
SOX5 is over-expressed in lung adenocarcinoma and is associated with *in vitro* cell invasion (**A**) Western blot analysis of SOX5 levels (top) in four human lung adenocarcinoma patients (LAC) and their respective non-tumor counterparts (N); Quantitative RT-PCR analysis of *SOX5*mRNA level (bottom) in the same patients, normalized versus GAPDH; ^***^:*p* < 0.001 (LAC *vs.* N). (**B**) Immunohistochemical analysis of SOX5expression and localization in LACs and paracancerous tissues (Microscope magnification: 200×). SOX5 was localized in the cytoplasm. (**C**) Western blot analysis of SOX5 level (top) in several lung cancer cell lines and bronchial epithelium cell line (16HBE); Quantitative RT-PCR analysis of *SOX5* level in these cell lines (middle); Invasive capacity analysis in different cell lines (bottom), tested in the 8μm invasive chamber. Data represent mean ± SD calculated from triplicate experiments.

We first analyzed SOX5 expression in four human lung adenocarcinoma (LAC) samples and a series of cell lines. Quantitative RT-PCR analysis showed that transcriptional expression of SOX5 is higher in tumors than the paired non-tumor controls. Western blot also showed that SOX5 is over-expressed in tumor tissues, whereas it was weakly expressed in respective non-tumor controls (Figure 1A).

To verify SOX5 levels in LAC patients, 90 pairs of LAC tissues were examined by IHC and data were analyzed by SPSS software. Analysis revealed that SOX5 was frequently expressed in LACs, with only six cases (6.67%) negative for SOX5. We also found that 77 of 90 (85.55%) LACs had high SOX5 expression (Score 4 and Score 5) (Table 1). SOX5 protein was localized in the cytoplasm in all LAC cells and paracancerous tissues. Moreover, SOX5 expression in adjacent non-tumor tissues was lower than in LAC tissues (P< 0.0001) (Figure 1B, Table 2). According to our SOX5 expression scores, LAC tissue displays a positive correlation with paracancerous tissues (P<0.05), suggesting that SOX5 may perform similar biological functions in tumor tissue and tumor microenvironment (Supplementary Table 1).

**Table 1:** Description of the population studied by immunohistochemistry

Variables	*n* = 90
**Age (years)**	
<= 60	41(45.55%)
> 60	49(54.44%)
**Sex**	
Male	49 (54.44%)
Female	41 (45.55%)
**Tumour size**	
<= 5cm	74(82.22%)
> 5cm	16(17.78%)
**Histologic grade**	
I	3(3.33%)
II	66(73.33%)
III	21(23.33%)
**Tumour stage (T)**	
I	17 (18.89%)
II	50 (55.55%)
III	17 (18.89%)
IV	6 (6.67%)
**Lymph node metastases (N)**	
N0	39 (43.33%)
N1	17 (18.89%)
N2	15 (16.67%)
N3	4 (4.44%)
Nx	12(4.58%)
Unknown	3(3.33%)
**Distance metastases (M)**	
M0	88 (97.78%)
M1	1 (1.11%)
Unknown	1 (1.11%)
**SOX5 positive incidence**	
0	6 (6.67%)
1 (1%-20%)	0 (0%)
2 (21%-40%)	3 (3.33%)
3 (41%-60%)	2 (2.22%)
4 (61%-80%)	20 (22.22%)
5 (81%-100%)	57(63.33%)
Unknown	2(2.22%)
**SOX5 Staining intensity**	
0-1 (including 0)	22(24.44%)
1-2 (including 1)	48(53.33%)
2-3 (including 2)	15(16.67%)
3	3(3.33%)
Unknown	2(2.22%)

**Table 2:** SOX5 expression difference analysis (Npar paired analysis)

Sample	Mean ± Std. Deviation	Number	*P*-value
Lung adenocarcinoma tissues	5.952 **±** 3.516	88	0.000^***^
Adjacent mucosa	1.591 **±** 1.238	88	

^***^:*P* < 0.001.

Comparison of SOX5 expression with the *in vitro* invasive capacity of lung carcinoma cell lines and bronchial epithelium cell line (16HBE) revealed that SOX5 mRNA and protein levels are positively correlated with cell invasive capacity (Figure 1C). 16HBE *SOX5* mRNA level was too low to detect. All of these observations indicate that SOX5 is over-expressed in lung adenocarcinoma and promotes tumor progression.

### SOX5 expression is correlated with poor prognosis in LAC patients

To investigate the clinicopathological and prognostic significance of SOX5 expression in LAC patients, immunohistochemical staining index was analyzed. It showed that SOX5 expression in lung adenocarcinoma was closely associated with clinical stages (r = 0.254, *P* < 0.05), and that SOX5expression in paracancerous tissues was correlated with tumor size (r = 0.211, P < 0.05) (Table 3). No other significant relationships between SOX5 expression and clinicopathological features was observed.

**Table 3:** Spearman’s correlation analysis between SOX5 expression and clinicopathological features

			Sex	Age	Tumor size	Pathological grade	T	N	M	Clinical stage
Spearman’s rho	SOX5 grouping in cancerous tissues	Correlation Coefficient	-.103	-.030	.092	.166	.124	.169	.043	.254^*^
		Sig. (2-tailed)	.341	.780	.394	.121	.251	.122	.692	.029^*^
		N	88	88	88	88	88	85	87	**74**
	SOX5 grouping in paracanerous tissues	Correlation Coefficient	-.017	-.091	.166	.116	-.019	-.003	-.059	.018
		Sig. (2-tailed)	.873	.397	.122	.284	.862	.976	.588	.882
		N	88	88	88	88	88	85	87	74
	SOX5 score in cancerous tissues	Correlation Coefficient	.031	.058	.126	-.048	-.171	-.052	.067	-.077
		Sig. (2-tailed)	.772	.590	.241	.654	.112	.639	.536	.514
		N	88	88	88	88	88	85	87	74
	SOX5 score in paracanerous tissue	Correlation Coefficient	-.128	-.056	.211^*^	.114	-.023	.026	-.049	.096
		Sig. (2-tailed)	.236	.603	.049^*^	.292	.829	.814	.653	.418
		N	88	88	**88**	88	88	85	87	74

**Score and grouping:**

Score of staining intensity: 0 = Score 0 , 1= Score 1, 2= Score 2, 3 = Score 3;

Score of positive incidence: 0% = Score 0, 1%-20% = Score 1, 21%–40% = Score 2, 41%-60% = Score 3, 61%–80% = Score 4, 81%-100%=Score 5;

Total Score= “Score of staining intensity” plus “Score of positive incidence”;

Grouping: if the Total Score ≤ 2, then the sample divided in the “SOX5 low expression group”; if Total Score > 2, the sample divided in the “SOX5 high expression group”.

SOX5 grouping in cancerous tissues has positive correlation with clinical stage (Correlation Coefficient = 0.254, ^*^ for *P* < 0.05).

SOX5 Score in paracancerous tissues has positive correlation with tumor size (Correlation Coefficient = 0.211, ^*^ for *P* < 0.05).

**Figure 2:**
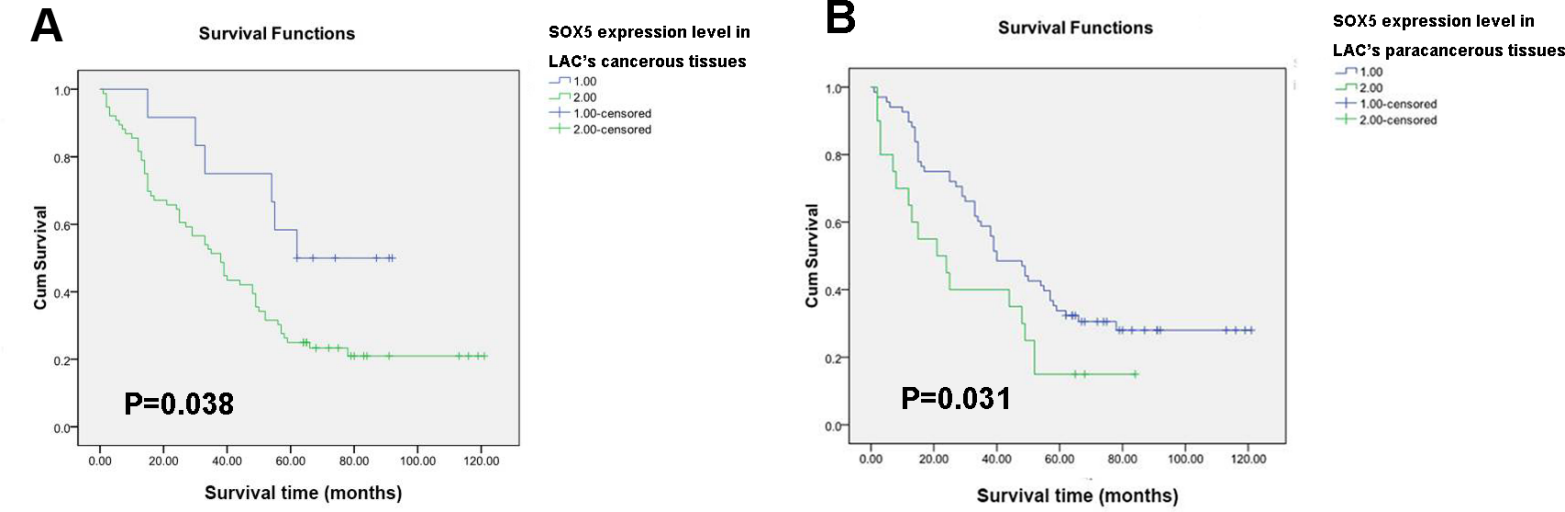
SOX5 (in both tumor and paracancerous tissues) correlates negatively with survival in LAC patients (**A**) Overall survival rate presented in Kaplan−Meier survival curve for cases with high SOX5 expression versus cases with low SOX5 expression in 90 LAC patients’ cancerous tissues. There was significant difference in prognosis between these two groups (*P* < 0.05) (**B**) Kaplan−Meier survival curve analysis of SOX5 expression in the 90 LAC patients’ paracancerous tissues (*P* < 0.05).

Survival single-factor analysis was examined with Kaplan-Meier analysis and the log-rank test (Supplementary Tables 2, 3). Patients with lower SOX5 expression in LAC tissues had longer survival time than those with high SOX5 expression (*P* < 0.05), and the same correlation was found in adjacent non-tumor tissues (*P* < 0.05). Kaplan Meier curve showed a negative correlation between high SOX5 expression and overall survival (OS) in both LAC tissues and the paired adjacent tissues (Figure 2A, 2B). Univariate and multivariate analyses showed that high SOX5 expression in adjacent non-tumor tissues was an independent prognostic factor for poor survival of LAC patients (*P* <0.05) (Supplementary Table 4).

### Down-regulation of SOX5 attenuated lung cancer cell growth and metastasis

**Figure 3:**
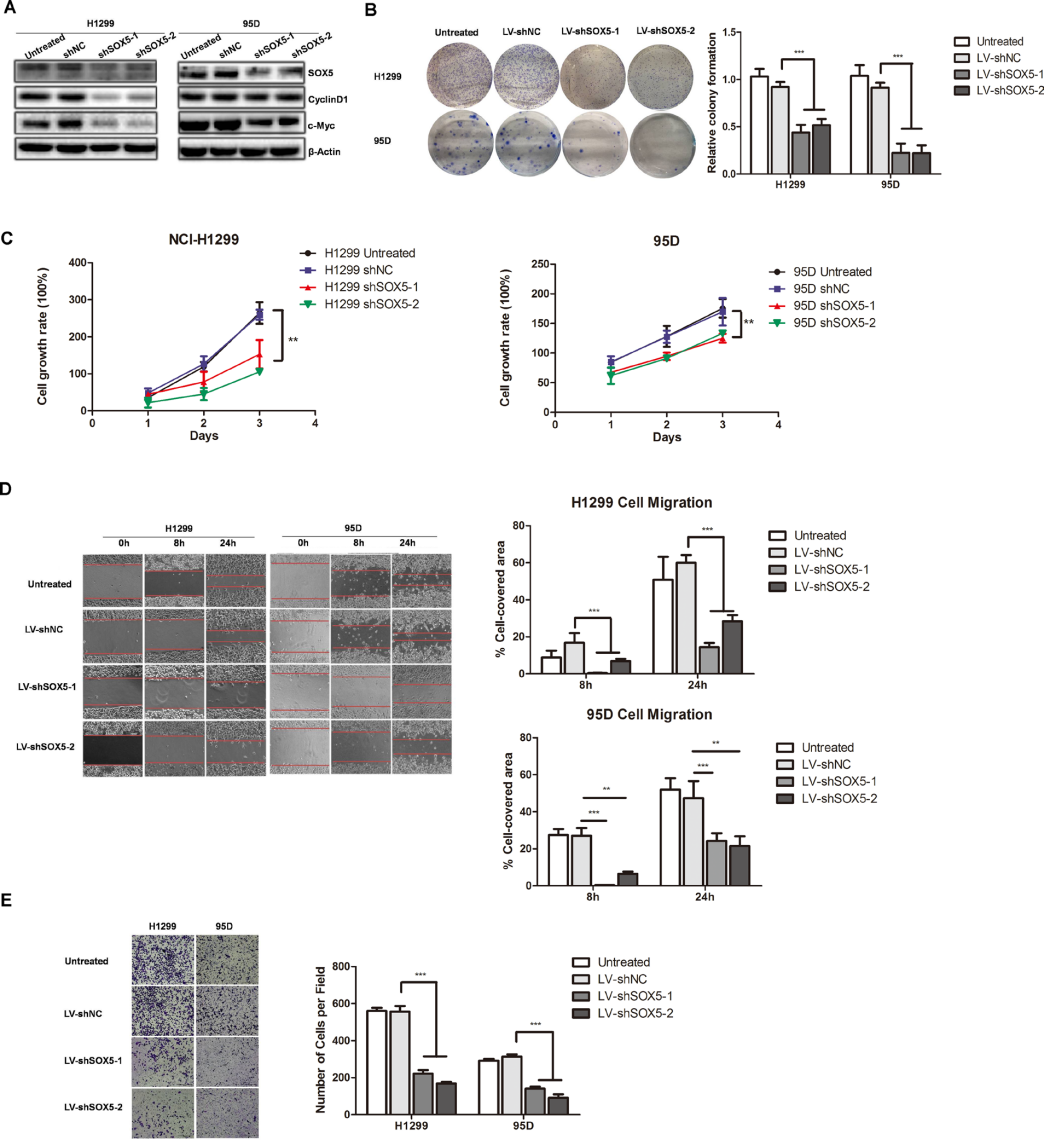
SOX5 knockdown inhibits LAC cell proliferation and metastasis *in vitro* (**A**) SOX5 expression in control (Untreated), sh Negative Control (scramble sequence, NC), and SOX5 knockdown (shSOX5-1and shSOX5-2) LAC cells H1299 and 95D was detected by western blot. Cell cycle biomarkers CyclinD1 and c-Myc were also detected. (**B**) Colony formation analysis of shSOX5-1 and shSOX5-2, as well as untreated and negative control cells. Cells with stable shRNA expression were seeded into 6-well plates at 2000 cells per well. Seven days later, crystal violet staining and colony counting was performed. Data were shown on the right as mean ± SD and ^***^ for *P* < 0.001 (LV-shNC *vs.* LV-shSOX5). (**C**) Effect of SOX5 knockdown on cell proliferation was determined by CCK8 assay at the indicated time point. ^**^ for *P* < 0.01(LV-shNC *vs.* LV-shSOX5). (**D**) Effect of SOX5 knockdown on cell migration by wound-healing assay. Statistical analysis was done with GraphPad Prism 5, ^**^ for *P* < 0.01 and ^***^ for *P* < 0.001 (LV-shNC *vs.* LV-shSOX5). (**E**) Invasive transwell analysis of shSOX5-1 and shSOX5-2, as well as untreated and shNC cells. ^***^ for *P* < 0.001 (LV-shNC *vs.* LV-shSOX5).

Given that SOX5 was up-regulated in LAC, we explored the function of SOX5 in LAC cell lines. Using lentivirus shRNA, we silenced SOX5 expression in NCI-H1299 and 95D cells, which had relatively high endogenous SOX5 expression (Figure 1C). Successful depletion of endogenous SOX5 expression was confirmed by Western blot. Cell proliferation biomarkers, such as CyclinD1 and c-Myc, were down-regulated when SOX5 was depleted in NCI-H1299 and 95D cells (Figure 3A). Colony formation assay showed that shSOX5-1 and shSOX5-2 cells formed smaller and fewer colonies than control cells (Figure 3B). CCK8 assay also revealed that shSOX5-1 and shSOX5-2 cells grew much slower than control cells (Figure 3C). Furthermore, wound healing assay and transwell assay showed that SOX5 inhibition impeded cell migration and invasion in LAC cells (Figure 3D, 3E). These results indicate that silencing SOX5 expression can inhibit LAC cell proliferation, migration, and invasion *in vitro*.

### SOX5 up-regulation promotes LAC cell proliferation, migration, and invasion *in vitro*

**Figure 4:**
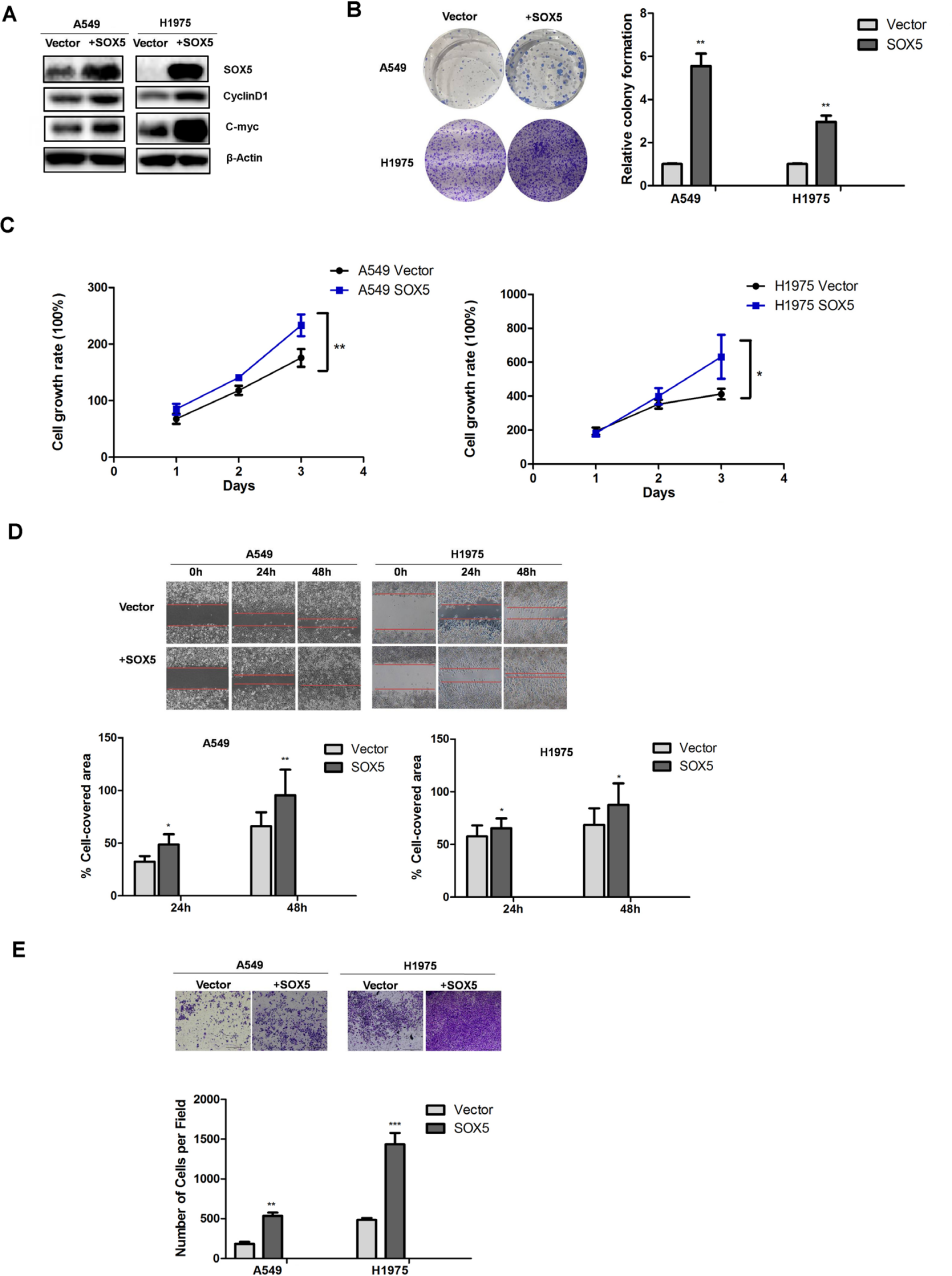
Effects of SOX5 overexpression on LAC cell proliferation and metastasis (**A**) SOX5 expression in control (Vector) and SOX5 overexpression (SOX5) in A549 and H1975 cells were detected by western blot. CyclinD1 and c-Myc were also detected. (**B**) Effect of SOX5 overexpression on colony formation. A549 and H1975 cells with stable expression of vector or SOX5 were seeded into 6-well plates at 2000 cells per well and cultured for 7 days, followed by crystal violet staining and colony counting. Data were analyzed as mean ± SD and ^**^ for *P* < 0.01(Right, Vector *vs.* SOX5). (**C**) Effect of SOX5 overexpression on the cell proliferation rate in A549 and H1975 cells expressing empty vector or SOX5 and determined by CCK8 assay at the indicated time point, ^*^ for *P* < 0.05 and ^**^ for *P* < 0.01(Vector *vs.* SOX5). (**D**) Effect of SOX5 overexpression on cell migration in the wound-healing assay. Statistical analysis was done with GraphPad Prism 5, ^*^ for *P* < 0.05 and ^**^ for *P* < 0.01(Vector *vs.* SOX5). (**E**) Invasive transwell analysis of SOX5 overexpression in A549 and H1975 cells. ^**^ for *P* < 0.01(Vector *vs.* SOX5).

To further determine whether SOX5 affected proliferation, migration, and invasion of LAC cells, we stably transfected lentivirus with an over-expression SOX5 gene into A549 and H1975 cells which have relatively low SOX5 levels. Western blot was used to demonstrate SOX5 over-expression (Figure 4A). Western blot for cell proliferation markers, colony formation assay, CCK8 assay, wound healing assay, and cell invasion assay reconfirmed that SOX5 over-expression promoted LAC cell proliferation, migration, and invasion (Figure 4A–4E).

### SOX5 facilitates EMT in lung adenocarcinoma

**Figure 5:**
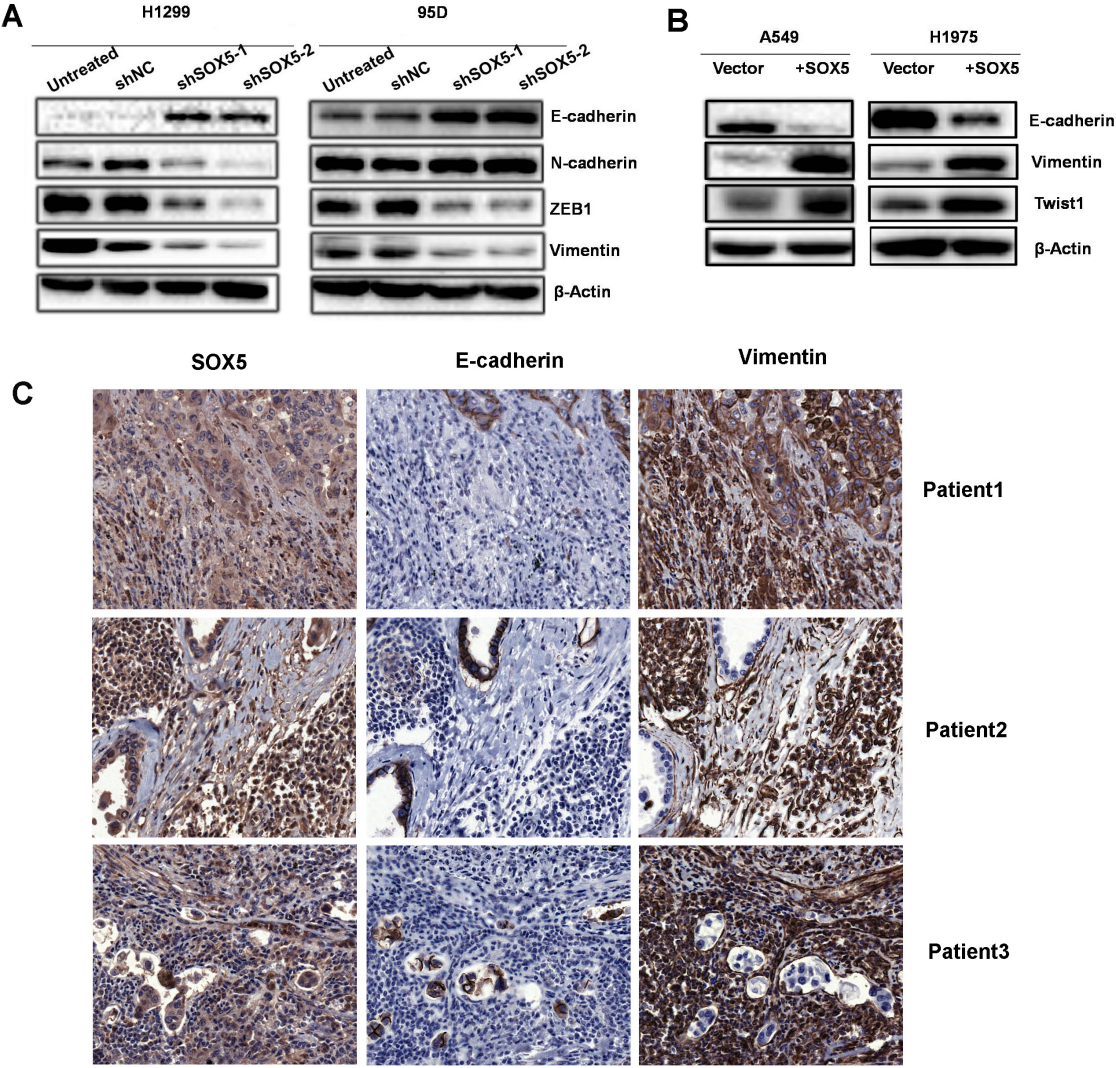
SOX5 facilitates the epithelial-mesenchymal transition (EMT) in lung adenocarcinoma (**A**) Western blot analysis of epithelial (E-cadherin) and mesenchymal (N-cadherin , ZEB1, Vimentin) markers in control (Untreated), sh Negative Control (scramble sequence, NC) and SOX5 knockdown (shSOX5-1and shSOX5-2) LAC cells H1299 and 95D. (**B**) Western blot analysis of epithelial (E-cadherin) and mesenchymal (Vimentin and Twist1) markers in A549 and H1975 cells (lentivirus with empty vector or SOX5 transfected). (**C**) Immunohistochemical analysis of SOX5, E-cadherin, and Vimentin expression in three advanced-stage LAC patients with serial sections.

To investigate the effects of SOX5 on the EMT process, the protein expression of mesenchymal phenotype cell biomarkers (ZEB1, Vimentin, N-cadherin, and Twist1) and epithelial phenotype cell biomarker (E-cadherin) were measured by western blot in SOX5-silenced cells, over-expression cells, and corresponding control cells (Figure 5A, 5B). SOX5 depletion attenuated mesenchymal biomarkers including ZEB1, Vimentin, and N-cadherin, while epithelial marker E-cadherin was up-regulated. SOX5 over-expression was followed by increased Vimentin and twist1 levels, while E-cadherin levels decreased. Serial sections of tissues from three late stage LAC patients were stained with SOX5, E-cadherin, and Vimentin. SOX5 expression in LAC increases Vimentin expression and decreased E-cadherin expression (Figure 5C).

### SOX5 knockdown in LAC cells inhibits their proliferation and metastasis in the zebrafish model

**Figure 6:**
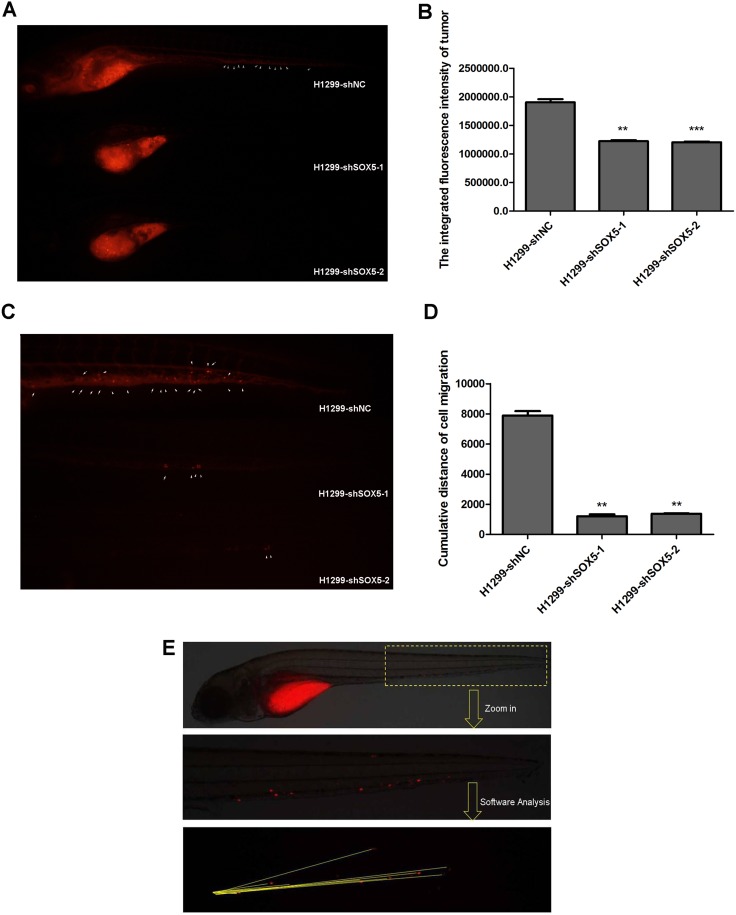
Silencing SOX5 reducesH1299 cell proliferation and metastasis *in vivo* (**A**) Zebrafish cancer xenograft assay of H1299 cells with silenced SOX5. Arrows indicate metastatic tumor cells. (**B**) The integrated fluorescence intensity of tumors in the H1299-shSOX5 group compared with the H1299-NC group,^**^p < 0.01,^***^p < 0.001. (**C**) The metastasis inhibition of SOX5 knockdown. Arrows indicate metastatic tumor cells. (**D**) The cumulative distance of cell migration in the H1299-shSOX5 group compared with the H1299-NC group, ^**^p < 0.01. (**E**) The schematic diagram of data collection and analysis in the zebrafish cancer xenograft model.

We further analyzed SOX5 silencing *in vivo* using a zebrafish xenograft model, which has been used in cancer proliferation and metastasis analyses [23]. Using the zebrafish embryo xenograft model, H1299 cells stably transfected with lentivirus shRNA targeting *SOX5* (shSOX5), or the empty vector (NC), were examined for cell proliferation and metastasis. SOX5 knockdown reduced cancer cell proliferation three days post-inoculation (Figure 6A, 6B). The inhibitory rates of tumor growth in H1299 shSOX5-1 and H1299 shSOX5-2 were 36% (*P* < 0.01) and 37%, respectively (*P* < 0.001) (Table 4). Cumulative distance of cell migration was calculated to measure metastasis. The H1299-NC group had 100% metastasis-positive fish, while shSOX5-1 and shSOX5-2 groups had 30% metastasis-positive fish. This indicates SOX5 inhibition can reduce the incidence rate of LAC metastasis. Based on the *t* test, the ability to metastasize was higher in the H1299-NC group (7889 pixels) than in the two shSOX5 groups (1206 pixels and 1370 pixels, respectively) (*p* < 0.01) (Figure 6C, 6D). The cumulative distance of cell migration was analyzed as a schematic diagram showed in Figure 6E. The inhibitory rate of metastasis was 85% and 83% in the shSOX5 groups, respectively (Table 5).

**Table 4:** The inhibitory rates of tumor growth in zebrafish xenograft model (mean ± SE, *n* = 10)

Groups	The integrated density of fluorescence (pixel)	The inhibitory rates of tumor growth(%)
H1299NC	1905062 ± 168819	−
H1299KD1	1224250 ± 61362^**^	36^**^
H1299KD2	1204308 ± 43719^***^	37^***^

Compared with H1299NC group, ^**^p < 0.001, ^***^p < 0.001.

**Table 5:** The inhibitory rate of metastasis in zebrafish xenograft model (mean ± SE)

Groups	The cumulative distance of cell migration (pixel)	The inhibitory rate of metastasis (%)
H1299NC	7889 ± 919	-
H1299KD1	1206 ± 386^**^	85^**^
H1299KD2	1370 ± 91^**^	83^**^

H1299NC group: *n* = 10, H1299KD1/H1299KD2 group: *n* = 3, Compared with H1299NC group, ^**^p < 0.01

## DISCUSSION

SOX5 is a member of the sex-determining region Y-related high mobility group box (SOX) transcription factor family, which consists of at least 20 highly conserved transcription factors in humans [24]. SOX family genes regulate cell fate, including cell development, homeostasis, and regeneration [25]. Several members of SOX family stimulate the initiation and progression of different cancers, including SOX2 and SOX4 [26, 27]. However, very few studies focused on the correlation of SOX5 and cancer.

In this study, we demonstrated that SOX5 was over-expressed in lung adenocarcinoma. We found that high SOX5 expression correlated to clinical stages and overall survival time in LAC patients. We also discovered that SOX5 levels in paracancerous tissues correlated to tumor size and poor prognosis. We conclude that SOX5 expression in adjacent non-tumor tissues promotes LAC tumorigenicity.

Silencing SOX5 in NCI-H1299 and 95D cells impeded cell proliferation and metastasis; increasing SOX5 in A549 and H1975 cells accelerated tumor progression. We found that SOX5 promotes cell proliferation and metastasis by inducing EMT in LAC. Silencing SOX5 in NCI-H1299 and 95D cells led to up-regulated epithelial markers and down-regulated mesenchymal markers; over-expressing SOX5 in A549 and H1975 cells led to up-regulated mesenchymal markers and down-regulated epithelial markers. The same was found in SOX5 over-expressing LAC tissues.

Our findings suggest that SOX5 promotes tumor metastasis and could be a novel diagnostic marker and potential therapeutic and prognostic target in lung adenocarcinoma.

## MATERIALS AND METHODS

### Patient information and tissue specimens

The paraffin-embedded samples from patients investigated in this study were collected retrospectively from archival material stored in the biobank center at the National Engineering Center for Biochip in Shanghai. Samples came from 90 patients who had undergone section surgery between 2004 and 2009. Written informed consents for the tissue specimens were received from all participants, and the study was approved by the ethical committee of biobank center related hospitals.

Clinicopathological data was obtained from original pathology reports, including gender, age, tumor size, grade of pathological classification, tumor location, invasion, LN metastases, tumor metastases, and clinical tumor stage. Staging of lung adenocarcinoma were assessed according to the American Joint Committee on Cancer (AJCC) criteria. A detailed description of clinical and pathological data of these 90 patients is provided in Table 1.

The follow-up period was from the date of surgery to the patient’s death from lung adenocarcinoma. Two patients were lost to follow-up and excluded from the 5-year survival analysis. Among the remaining 88 patients, 67 patients passed away during the follow-up period.

### Tissue microarray construction

A tissue microarray instrument was used to make holes with a diameter of 0.6 mm and a depth of 2 mm on the paraffin block. Based on the microscopic test of pathological sections through H&E staining, representative tumor and its adjacent lung adenocarcinoma tissues on the corresponding paraffin blocks were selected by a pathologist. The tissue mass was obtained through the fine hollow needles of the tissue microarray instrument, which was pressed into the paraffin block holes. Then, a second set of holes was constructed and arrayed in the tumor and its adjacent lung adenocarcinoma tissues. Serial sections (0.66 um thick) were made from the arrayed paraffin block and placed onto glass slides The tissue microarray was validated by two pathologists using H&E staining.

### Immunohistochemistry

Immunohistochemical analysis was performed to investigate SOX5 expression in LAC. Slides were deparaffinized in xylene for five minutes, three times. Slides were rinsed in 100% alcohol for three minutes, three times. This triple rinse was repeated with 95% alcohol, then the slides were placed in 70% alcohol for a single three minutes. Slides were transferred to dH_2_O before being rinsed three times in PBS, five minutes each time.

We then blocked endogenous peroxidase activity. Slides were incubated for 10minutes in 3% H_2_O_2_ in PBS, followed by a rinse with H_2_O. Slides were then immersed in Target retrieval solution (Dako, code S1699) and boiled in a pressure cooker for five minutes at a high temperature setting. After three, five-minute PBS rinses, the sections were incubated for 30minutes with Avidin/10% normal goat serum in PBS.

The sections were incubated with specific diluted antibodies with biotin solution overnight at 4°C. Primary antibodies included: SOX5 (Santa, sc9001), Vimentin (CST, 5741) and E-cadherin (CST, 3195). Slides were rinsed 3 times with PBS for 5 minutes each before incubating for 30 minutesin 1:200 diluted biotinylated secondary antibodies. After another triple PBS rinse for five minutes each, the sections were incubated for 30min with Vexta stain Elite ABC reagent (Vector, PK-6101). After a final triple PBS rinse for five minutes each, the sections were incubated in peroxidase substrate solution (Vector Sk-4100) until the desired stain intensity developed. The slide sections were rinsed in tap water, counterstained, then coverslipped. The tissue sections were observed and scanned with a3D Histech Pannoramic MIDI microscope (Hungary).

The IHC results were determined by two independent, blinded pathologists. The staining intensity of cancer cells was scored as 0, negative; 1, weak; 2, moderate; 3, strong. For statistical evaluation, tumors were scored as 0, non-staining; 1,1–20%; 2, 21–40%; 3, 41–60%; 4, 61–80%;5, 81–100% positive cells. The total histological score, which was the product of the intensity and percentage scores, was utilized to determine the result. A total histological score < 2 indicated low expression, and a total histological score ≥ 2 denoted high expression.

### Cell line and cell culture

Four lung adenocarcinoma cell lines (H1299, 95D, A549, H1975) and one bronchial epithelial cell line (16HBE) were obtained from Cell bank of Chinese Academy of Sciences (Shanghai, China) with STR verification. The 95D cells were cultured in DMEM (Gibco) with 10%FBS (Gibco). H1299, A549, H1975, and 16HBE cells were cultured in RPMI-1640 with 10%FBS. All cells were maintained at 37°C in a humidified incubator with 5% CO_2_.

### RNA isolation and quantitative RT-PCR

Total RNA was extracted from tissues or cells using TRIzol reagent (Invitrogen, California, USA), and reverse transcription was performed using the HiFiScript gDNA Removal cDNA Synthesis Kit (KangWeiShiJi, China) according to the manufacturer’s instructions. The quantification of gene transcripts was determined by quantitative RT-PCR using SYBR Premix Ex Taq II (Takara, China) and the 7900HT Fast Real-Time PCR System (Applied Biosystems). The primers were as follows, SOX5: 5′-ATAAAGCGTCCAATGAATGCCT-3′ and 5′-GCGAGATCCCAATATCTTGCTG-3′; GAPDH: 5′-ACAACTTTGGTATCGTGGAAGG-3′ and 5′-GCCATCACGCCACAGTTTC-3′. The results were analyzed using SDS 2.2.2 software (Applied Biosystems) and recorded as threshold cycle (Ct) values. The relative mRNA levels of the targeted gene were adjusted according to housekeeping gene GAPDH and determined as 2^-ΔCT^. The experiment was performed in triplicate.

### Western blotting

Cells were harvested, and the total protein was extracted and quantified using the BCA kit (Thermo, USA). Equal amounts of proteins were boiled in sample loading buffer, separated by electrophoresis on 10% SDS–PAGE gel, and transferred to PVDF membrane (Millipore, Bedford, MA, USA). Membranes were blocked in 5% skim milk powder in TBS-T (TBS plus 0.5% Tween-20) at room temperature, and the membranes were probed with primary antibodies at 4°C overnight. Primary antibodies included: SOX5 (1:1000, R&D systems, USA), E-cadherin, N-Cadherin, Vimentin, ZEB1, CyclinD1, c-Myc (1:1000, Cell Signaling Technology, USA), and β-Actin (1:3000, Sigma-Aldrich, USA). The membrane was then exposed to peroxidase-conjugated secondary antibody (1:5000, Lianke Bio, China). The signals were visualized by enhanced chemiluminescence kit (Millipore, USA) according to the manufacturer›s instructions. Anti-β-Actin antibody (Sigma-Aldrich) was used as a loading control.

### Lentivirus packaging and infection

Lentiviral short hairpin RNA (shRNAs) in hU6-MCS-Ubiquitin-EGFP-IRES-puromycin (Genechem, Shanghai,China) was used to express short hairpin RNA (shRNA). The RNAi sequences targeting to SOX5 gene were 5′- ACATATCAAAGAAGAGATA-3′ and 5′-ATGCAATGATGGATTTCAA-3′. Negative control sequence was 5′-TTCTCCGAACGTGTCACGT-3′. Lentivirus was produced in 293 T cells. LV-shSOX5 plasmids were transfected into HEK 293 T cells together with the lentiviral packaging vectors. Infection lentiviruses were collected 72 h after transfection and concentrated by ultracentrifugation using Beckman Instruments (Fullerton, CA, USA). H1299 and 95D cells were seeded in a six-well plate and infected with shSOX5-lentiviruse or NC-shRNA lentivirus, respectively, in 5 μg/ml polybrene. Stable transfected cells were selected with 2 μg/ml puromycin for 2 weeks followed by maintenance in 1 μg/ml puromycin. The knockdown efficiency was evaluated by fluorescence microscope and western blot.

The SOX5 overexpression lentiviral vector, pGC-FU-SOX5-3FLAG-SV40-EGFP-IRES-puromycin and the empty lentiviral vector for control were purchased from Genechem (China). The lentiviral vectors were co-transfected with helper plasmids Gag pol, VSVG into 293T cells. Then the supernatant was harvested at 72 hours post transfection and concentrated by the same method above. A549 cells were seeded in six-well plates and infected lentivirus with SOX5 and empty vector overnight in 5μg/ml polybrene. Stable cells selection method was performed as above. SOX5 overexpression was identified by western blot and fluorescence microscope.

### Colony formation assay

Stable cells were digested and seeded into six-well plates with 2000 cells per well in 2 ml complete medium. After incubating for 2 weeks, cells colonies were washed and fixed with 4% paraformaldehyde for 30 minutes at room temperature, followed by staining with 0.1% crystal violet. The number of colonies was counted under a fluorescence microscope. Each experiment was performed in triplicate and repeated three times. The Student’s *t*-test was used to evaluate statistical significance.

### Cell Counting Kit-8 (CCK8) assay

*In vitro* cell proliferation ability was determined by using the Cell Counting Kit-8 (CCK8; Sigma-Aldrich) assay according to the manufacturer’s protocol. Two thousand cells were plated overnight in 100 μL of culture medium into a well of 96-well plates. Four plates which are used for time course assessment are seeded at the same time. After culturing cells for an appropriate time (initial attachment, 24 h, 48 h, and 72 h), 10 μL of CCK-8 solution was added to each well. After 2h, the absorbance at 450 nm (OD450) was measured. The relative growth rate was adjusted by initial-attachment OD450, which was calculated as (OD450 of time point – OD450 of initial attachment)/ OD450 of initial attachment × 100%.

### Wound-healing assay

Wound-healing assay was used to observe migration ability. Briefly, cells were seeded into 6-well plates and formed a fluent monolayer. A vertical wound was made by dragging a plastic pipette tip across the cell surface, and detached cells were removed. Phase contrast images of the wounds were photographed at the time points of 0 h, 8 h, and 24 h. For each sample, at least three scratched fields were photographed.

### Cell invasion assay

Cells in 0.2 ml medium without FBS were placed on the top chamber of each insert (8 μm pore size, BD Biosciences, San Jose, CA, USA) with 40 μl of 1 mg/ml Matrigel. A total of 5× 10^4^ cells were suspended in 500 μL DMEM without serum and added to the upper chamber, while 750 μl DMEM containing 10% FBS added in the lower chamber. After 48 h of incubation, the cells on the upper layer were gently scratched with a cotton stick. The cells that invaded into the chamber membrane were fixed in 100% methanol for 30 minutes, then stained with 0.5% crystal violet. The membranes were then carved and embedded under cover slips. At least three random microscopic fields (magnification, 40×) were analyzed for each insert.

### Zebrafish cancer metastasis assay

The wild-type AB strain zebrafish (Danio rerio) were maintained at a density of 20 fish of both sexes per tank in an aquarium with a recirculating water system on a 14 h light/10 h dark cycle. Fish were fed with live brine shrimp or dry flakes. Two pairs of adult fish of 3 month old (2 males and 2 females) were separately maintained in a spawning tank and allowed to mate and spawn. The next day, embryos were collected and cultured at 28°C for 2-4 h. Embryos were maintained at 28°C in fish water (0.2% Instant Ocean Salt in deionized water, pH 6.9–7.2, conductivity 480∼510 μS/cm, and hardness 53.7∼71.6 mg/L CaCO_3_).

Adherent cancer cells (3 × 10^6^ cell/mL) were stained with 5μL/mL fluorescent Cell Tracker CM-Dil (Invitrogen) at 37°C for 5 min, followed by another incubation at 4°C for 15 min.CM-Dil labeled H1299 cells (800 cells per zebrafish) were transplanted into the yolk sac of 2 dpf normal wild type zebrafish by microinjection and maintained at 35°C to 3 dpf. The zebrafish with consistent xenografts were selected by microscope (30 fish in each group). The fish were incubated for another two days. Ten fish in each group were randomly selected and photographed using a Nikon AZ100 stereo fluorescence microscope at 80×magnification with the same exposure time for both the control and knockdown group. The integrated density of fluorescence was measured using Image-Pro Plus 6.0. The inhibitory effects of *SOX5* knockdown on human lung cancer xenograft growth in zebrafish were measured by the integrated fluorescence intensity (S). Inhibitory effects of *SOX5* knockdown on the metastasis of human lung cancer xenografts in zebrafish were measured by cumulative distance of cell migration (L).

### Statistical analysis

SPSS software (IBM, CA, USA) was used for patients’ tissues statistical analysis. SOX5 expression was compared between lung adenocarcinoma and paracancerous tissues using the non-parametric Mann-Whitney *U*-test. Overall survival was calculated and survival curves were plotted using the Kaplan-Meier method. The differences between groups were compared using log-rank test. Cox regression model was used to examine whether SOX5 and clinical features can serve as biomarkers of lung adenocarcinoma and paracancerous. A *P* value < 0.05 was considered statistically significant.

*In vitro* statistical analyses were performed with GraphPad Prism 5 software. Results of experiments were depicted as mean ± SD, and Student’s t test was used to determine differences between the two groups. A *P* value < 0.05 was considered statistically significant. ^*^P < 0.05; ^**^P < 0.01; ^***^P < 0.001.

## SUPPLEMENTARY MATERIALS TABLES



## Abbreviations

SOX5: Sex determining region Y-box protein
LAC: Lung Adenocarcinoma
EMT: Epithelial-mesenchymal transition
OS: Overall Survival
