# Supplementary material for: SOX5 predicts poor prognosis in lung adenocarcinoma and promotes tumor metastasis through epithelial-mesenchymal transition
Source: Oncotarget. 2017 Nov 6;9(13):10891–904. doi: 10.18632/oncotarget.22443 (PMC5834284; doi:10.18632/oncotarget.22443)
Supplement: Supplementary file 1 [file oncotarget-09-10891-s001.pdf]

SOX5 predicts poor prognosis in lung adenocarcinoma and promotes tumor metastasis through epithelial-mesenchymal transition

SUPPLEMENTARY MATERIALS

Supplementary Table 1: Spearman's correlation analysis between cancerous and paracancerous tissues

			S5 score in paracanerous tissue
Spearman's rho	S5 score in cancerous tissue	Correlation Coefficient	**.270***
		Sig. (2-tailed)	**.011***
		N	**87**

SOX5 in cancerous tissues and in paracancerous tissues have positive correlation (Correlation Coefficient = 0.270, *for $P < 0.05$).

Supplementary Table 2: Correlation analysis between SOX5 expression level in LACs and patients' survival

	Total N	N of Events	Censored	
			N	Percent
1.00 Low expression group	12	6	6	50.0%
2.00 High expression group	76	59	17	22.4%
Overall	88	65	23	26.1%

Overall Comparisons			
	Chi-Square	df	Sig.
Log Rank (Mantel-Cox)	4.286	1	**.038***

The log-rank test: * for $P < 0.05$

Supplementary Table 3: Correlation analysis between SOX5 expression level in matched adjacent non-tumor tissues and patients' survival

	Total N	N of Events	Censored	
			N	Percent
1.00 Low expression group	68	48	20	29.4%
2.00 High expression group	20	17	3	15.0%
Overall	88	65	23	26.1%

**Overall Comparisons**			
	Chi-Square	df	Sig.
Log Rank (Mantel-Cox)	4.675	1	**.031***

The log-rank test: * for $P < 0.05$

Supplementary Table 4: Cox regression analyses for contribution of various potential prognostic factors to survival in LAC patients

Variables in the Equation								
	**B**	**SE**	**Wald**	**df**	***p*-value**	**Exp(B)**	**95.0% CI for Exp(B)**	
							**Lower**	**Upper**
SOX5 score in cancerous tissues	.430	.457	.888	1	.346	1.538	.628	3.765
SOX5 score in paracanerous tissues	.897	.353	6.457	1	**.011***	2.452	1.228	4.899
Tumor size	.236	.364	.421	1	.516	1.266	.621	2.582
T	.184	.256	.515	1	.473	1.202	.727	1.986
N	.751	.446	2.843	1	.092	2.120	.885	5.077
Clinical stage	.236	.315	.565	1	.452	1.267	.684	2.347

SOX5 score in paracanerous tissues was recognized as an independent prognostic factor for poor survival of patients with LAC ($P = 0.011^{*}$)
